# Nanomaterial-Enhanced Corneal Cross-Linking: Engineering Strategies for Transforming Keratoconus Management

**DOI:** 10.3390/pharmaceutics18070778

**Published:** 2026-06-25

**Authors:** Liqin Huang, Yao Fu, Fang Li

**Affiliations:** 1 Department of Ophthalmology, Shanghai Ninth People’s Hospital, Shanghai Jiao Tong University School of Medicine, Shanghai 200011, China; huangliqin@sjtu.edu.cn; 2 College of Health Science and Technology, Shanghai Jiao Tong University School of Medicine, Shanghai 200025, China

**Keywords:** keratoconus, corneal cross-linking, transepithelial, nanomaterial, theranostics, artificial intelligence

## Abstract

Keratoconus, a progressive corneal ectasia, remains a major cause of irreversible visual impairment worldwide. Conventional corneal cross-linking (CXL) with riboflavin/ultraviolet A (UVA) has revolutionized clinical management, yet its efficacy is still constrained by epithelial barriers, oxygen dependence, and safety concerns in thin corneas. Emerging nanotechnology provides a transformative opportunity to overcome these bottlenecks. This review highlights the enhancement of riboflavin delivery efficiency by nanocarriers, the photodynamic optimization of nano-enhanced cross-linking agents, and the synergistic strengthening effect of nanocomposites on corneal mechanical strength. We emphasize not only their potential to enhance drug penetration, improve cross-linking efficiency, and extend clinical indications, but also their role in advancing toward a new generation of personalized, intelligent, and minimally invasive corneal therapy. Finally, we discuss translational challenges, including manufacturing, long-term biosafety, and regulatory frameworks, and present a theoretical roadmap that integrates nanotechnology, real-time imaging, and artificial intelligence (AI)-assisted decision-making to achieve a closed-loop “sense–decide–act” therapeutic system. By situating nanomaterial-enhanced CXL within precision ophthalmology, this review highlights its capacity to redefine the standard of care for keratoconus and related ectatic disorders.

## 1. Introduction

Keratoconus is a bilateral progressive disease characterized by thinning and protrusion of the central or paracentral corneal stroma, giving rise to myopia, irregular astigmatism, corneal scarring, and mild to severe visual impairment [1]. Typically, the diagnosis is made during the first or second decade of life, and the condition gradually worsens over the subsequent two decades until the corneal anatomy stabilizes [2]. Reported keratoconus prevalence has increased from approximately 0.054% before the 1980s [3] to 0.138% in a 2020 meta-analysis [4]. This apparent rise, primarily driven by the widespread application of advanced diagnostic tools like corneal topography and tomography, indicates that keratoconus is no longer a rare disease.

The maximum load, stress, stiffness, and energy absorption capacity of keratoconus are significantly lower than those of normal corneas [5]. The progression of keratoconus is driven by a complex interplay of structural and enzymatic dysfunctions. Morphologically, the disruption of the highly ordered orthogonal collagen network severely compromises the biomechanical stability of the corneal stroma [6]. At the molecular level, this structural fragility is exacerbated by elevated collagenase expression that drives excessive collagen degradation [7], compounded by reduced lysyl oxidase (LOX) activity, which impairs the natural cross-linking of these fibers [8].

Until about two decades ago, treatment for keratoconus was restricted to eyeglasses, rigid gas-permeable (RGP) contact lenses, and corneal transplants for patients with severe cases to restore vision. However, these methods could not inhibit the progression of the disease. The advent of corneal cross-linking (CXL) brought new hope. The procedure primarily aims to strengthen the cornea’s weakened stiffness and enhance its resistance to degradative enzymes, thereby halting the disease’s progression and preventing further vision loss. The Dresden standard protocol for CXL surgery first entered clinical practice in 2003. The protocol involves removing the central 7 mm of the corneal epithelium and instilling a 0.1% riboflavin solution at 5 min intervals over a 30 min period. During this time, the cornea is irradiated with ultraviolet A (UVA) light (wavelength 370 nm, intensity 3 mW/cm^2^) for 30 min, delivering a total dose of 5.4 J/cm^2^ to the cornea [9].

Despite being recognized as a milestone in keratoconus management, the procedural constraints of the Dresden protocol have driven the pursuit of next-generation, technology-enabled therapeutic modalities. Over the past two decades, the field has witnessed the development of various modified modalities, including accelerated CXL (A-CXL), epithelium-on CXL (epi-on CXL), and customized protocols for thin corneas. While various modified protocols have been developed to overcome the initial drawbacks of standard CXL, they often merely substitute one challenge for another. Pressing issues remain unresolved, including the formidable epithelial barrier limiting transepithelial efficacy, the oxygen dependency and high-energy demands of A-CXL, and the procedural constraints of thin-cornea protocols. Consequently, these traditional modifications still fall short of achieving an optimal balance between biomechanical efficacy and clinical safety. In recent years, nanotechnology has significantly advanced precision ophthalmology. Engineered nanomaterials such as liposomes for dry eye syndrome and targeted nanocarriers for posterior segment diseases like age-related macular degeneration (AMD) and glaucoma have proven effective in bypassing ocular barriers, prolonging drug residence time, and minimizing off-target toxicity [10,11,12]. Building on these clinical successes, applying materials science to corneal biomechanics offers a promising new direction. The unique physicochemical attributes of nanocarriers, such as their ultra-small size, tunable surface charge, and stimuli-responsive release kinetics, are well-positioned to address the specific bottlenecks of standard CXL. For instance, customized nanovehicles can facilitate the transport of large hydrophilic riboflavin molecules across the intact lipophilic epithelium [13], while advanced catalytic nanoplatforms can generate in situ oxygen to alleviate the hypoxic microenvironment in accelerated protocols [14]. Ultimately, integrating nanomaterials into CXL strategies provides a viable pathway to overcome these long-standing challenges, pushing the treatment toward safer, less invasive, and more personalized clinical outcomes.

### Terminology and Benchmark Standardization

To navigate the diverse nano-enabled strategies discussed herein, strict terminological boundaries are established. Traditional epithelium-off (epi-off) CXL, specifically the standard Dresden epi-off protocol (3 mW/cm^2^ UVA for 30 min, total 5.4 J/cm^2^), involves complete epithelial debridement prior to riboflavin and UVA application. Crucially, throughout this review, whenever the primary studies include the standard epi-off Dresden protocol as a control, it serves as the definitive benchmark for evaluating the therapeutic efficacy and safety profiles of emerging nanotechnologies. A-CXL refers to protocols employing higher UVA irradiance with shortened exposure times, exemplified by the standard accelerated epi-off protocol (9 mW/cm^2^ UVA for 10 min, total 5.4 J/cm^2^). The terms epi-on and transepithelial are used interchangeably to describe non-invasive protocols that preserve the epithelial barrier. Within this category, iontophoresis-assisted CXL (I-CXL) specifically denotes the application of electrical gradients to actively drive charged formulations across the intact cornea. Finally, customized CXL encompasses individualized irradiation parameters and spatially tailored interventions designed to meet patient-specific biomechanical stiffening requirements.

## 2. Current Challenges in CXL

### 2.1. Transepithelial Riboflavin Permeation Barrier

In the standard Dresden protocol, mandatory epithelial debridement not only underlies major postoperative complications such as pain, haze, keratitis, and sterile infiltrates but also renders standard CXL anatomically unsuitable for inherently thin corneas. Epi-on CXL circumvents these clinical complications by maintaining epithelial integrity. Large-scale clinical evaluations consistently show that while epi-on CXL offers a superior safety profile, its biomechanical efficacy remains suboptimal compared to the standard epi-off technique [15]. This efficacy disparity stems primarily from the robust barrier function of the intact epithelium, as the substantial molecular size of riboflavin hinders paracellular transport through tight junctions, while the layer’s hydrophobic nature further repels the hydrophilic photosensitizer [16].

To improve riboflavin penetration and narrow the gap between epi-on and conventional epi-off CXL, two principal strategies have been developed. First, I-CXL applies a low electrical current (0.5–1 mA) to facilitate the penetration of negatively charged riboflavin through the intact epithelium [17]. Prevailing evidence indicates that I-CXL achieves better outcomes than conventional epi-on CXL but is less effective than the epi-off standard [18,19]. Second, penetration enhancers use chemical agents to degrade intercellular tight junctions and permit riboflavin passage [20]. These agents transiently alter membrane structure or function to increase drug transport across ocular surface barriers [21]. Commonly used agents include benzalkonium chloride (BAC) [22], tetracaine [23], ethanol [24], and vitamin E–tocopherol polyethylene glycol 1000 succinate (VE-TPGS) [25]. However, their use may compromise epithelial barrier integrity, so permeation efficiency must be balanced against safety (e.g., irritation and long-term toxicity) [26].

### 2.2. Oxygen Supply Limitation in A-CXL

Oxygen is a prerequisite for initiating adequate cross-linking reactions [27]. Given that oxygen consumption positively correlates with UVA intensity, the elevated irradiation necessitated by A-CXL protocols accelerates stromal oxygen depletion, thereby compromising biomechanical efficacy [28]. This oxygen deficit represents a fundamental mechanism behind the failure of the reciprocity law, explaining why A-CXL often fails to elicit a hardening effect comparable to the standard protocol despite equivalent UVA fluence. Consequently, implementing enhanced oxygen management, such as supplemental oxygen, is imperative at higher irradiance levels to mitigate hypoxia and ensure robust cross-linking [29].

Several physical strategies have been engineered to artificially boost external oxygen concentration during CXL. Devices, including specialized eyelid speculums [30], suspended polycarbonate tubes [31], and dedicated oxygen masks [32], all aim to maintain a continuous hyperoxic environment over the ocular surface. Nevertheless, one study reported comparable biomechanical strengthening during high-intensity CXL regardless of oxygen supplementation, a limitation likely stemming from the slow passive diffusion of oxygen into the stroma combined with the drastically shortened exposure time of A-CXL [33].

Another strategy to circumvent the hypoxic bottleneck involves pulsed UVA irradiation, which utilizes alternating exposure cycles to provide critical temporal windows for stromal reoxygenation. This modality is kinetically justified by the rapid depletion of stromal oxygen within the initial 15 s of continuous irradiation, followed by its efficient replenishment during the unexposed intervals [28]. Evidence from multiple studies suggests that accelerated pulsed high-fluence CXL is safe and effective for progressive keratoconus [34,35,36] and is applicable to pediatric cases [37]. However, a meta-analysis has not demonstrated a significant advantage over the standard protocol [38].

### 2.3. Corneal Thickness Limitation

A major limitation of the Dresden protocol is that it cannot be applied to corneas with stromal thickness <400 µm. This restriction is primarily dictated by the vulnerability of the underlying corneal endothelium to UVA irradiation, as excessive exposure can trigger irreversible endothelial toxicity and subsequent corneal edema. Unfortunately, patients with advanced keratoconus often present with thin corneas [39].

To circumvent this anatomical constraint, several strategies have been engineered to artificially augment corneal thickness prior to irradiation. Iatrogenic stromal swelling via hypo-osmolar riboflavin expands clinical eligibility by inducing a safe pachymetric buffer [40] and can yield anterior biomechanical strengthening comparable to standard formulations [41]. However, in extremely thin corneas (<330 µm), the resulting cross-linked depth often proves insufficient to arrest ectatic progression [42]. Alternative physical shielding techniques have also been developed to protect the endothelium, though they invariably trade biomechanical robustness for safety. For instance, contact lens-assisted CXL (CACXL) provides a reliable artificial barrier [43], but this mechanical addition reduces stromal stiffening by approximately one-third [44]. Similarly, customized epithelial-island protocols (EI-CXL) utilize localized epithelial preservation [45], which subsequently restricts oxygen diffusion, yielding an irregular, shallower demarcation line and an attenuated cross-linking response [46,47]. Because these anatomical workarounds inherently compromise treatment efficacy, modern therapeutic paradigms are increasingly shifting away from artificially altering the tissue toward customizing irradiation parameters to match the baseline corneal profile.

## 3. Emerging Nanotechnology Paradigms in CXL

This section maps emerging nanotechnologies directly to the clinical bottlenecks identified in Section 2. To overcome the epithelial barrier (Section 2.1), Section 3.1 evaluates delivery platforms engineered for transepithelial photosensitizer penetration. To address stromal hypoxia in accelerated protocols (Section 2.2), Section 3.2 examines nanomaterials engineered for in situ oxygen generation and oxygen-carrying microneedles. To bypass corneal thickness constraints and endothelial vulnerability (Section 2.3), Section 3.3 introduces visible-light paradigms and advanced two-photon strategies. Finally, Section 3.4 expands the therapeutic repertoire to multimodal pharmacological interventions. Collectively, these targeted solutions establish the technical foundation for the AI-driven, closed-loop theranostic framework envisioned in Section 4.

### 3.1. Advanced Delivery Platforms for Stromal Penetration

#### 3.1.1. Emulsified and Lipid-Based Nanosystems

As early-stage preclinical platforms, emulsified nanosystems overcome the biphasic corneal barrier by physically partitioning hydrophilic photosensitizers into a lipophilic phase, an approach driven by their inherent amphiphilic nature and thermodynamic stability [48,49]. Mechanistically, these systems utilize surfactants and positive surface charges to promote electrostatic adhesion to the mucosal layer. A clear demonstration of this principle is nanoemulsion-formulated riboflavin-5′-phosphate assessed in an ex vivo rabbit model. Pharmacokinetic evaluations reveal that this formulation drives transepithelial permeation to such an extent that, after 240 min, stromal riboflavin concentrations actually exceed the standard epi-off Dresden protocol [50]. While this indicates excellent permeability, the extended exposure time and the absence of direct biomechanical stiffening data suggest that further parameter optimization is required for clinical translation. Alternatively, purely physicochemical characterizations have shown that water-dilutable microemulsions utilize a dilution-triggered release mechanism; the photosensitizer remains structurally constrained within the surfactant network at low water content until significant aqueous dilution at the ocular surface liberates the riboflavin for subsequent stromal penetration [51]. Yet, successful barrier penetration does not automatically guarantee optimal clinical efficacy. For instance, an ex vivo evaluation of microemulsions demonstrated an 87.8% increase in corneal stress compared to untreated tissue; however, as the final biomechanical stiffening was not directly benchmarked against the standard epi-off Dresden protocol [52], its comparative therapeutic potential remains to be fully established.

Within established preclinical approaches, solid and vesicular lipid nanocarriers expand the ocular drug delivery repertoire but struggle directly with the inherent hydrophilicity of standard photosensitizers. Conventional liposomes yield riboflavin-5′-monophosphate encapsulation efficiencies of merely 3.5% to 41.8%, failing to secure the payload required for effective stromal saturation [53]. Pharmacokinetic testing in ex vivo bovine corneas demonstrated that hydrophilic molecules tend to rapidly escape from the aqueous core during ocular surface interaction, meaning liposomes fail to show a statistically significant permeation advantage over simple aqueous solutions. Nanostructured lipid carriers (NLCs) bypass this volume limitation by replacing the classic vesicular architecture with a highly disordered lipid matrix capable of trapping significantly larger aqueous fractions [54]. To optimize transepithelial flux, researchers have explored two distinct NLC modification strategies: incorporating the chemical enhancer Transcutol P to transiently relax epithelial tight junctions or engineering cationic NLCs featuring a zeta potential of +16.63 to +18.8 mV to promote electrostatic adhesion with the negatively charged mucosal layer [55]. When evaluated in in vivo rat and ex vivo rabbit models, the optimized enhancer-loaded system yielded significantly higher corneal stress at 10% strain than the traditional commercial epi-off control MedioCROSS D and matched the efficacy of the transepithelial alternative MedioCROSS TE without inducing epithelial injury.

#### 3.1.2. Polymeric Nanoparticles and Hydrogels

Operating primarily as versatile in vitro and ex vivo preclinical platforms, polymeric nanoparticles (NPs) provide a distinct mucosal advantage by utilizing polymer-specific bioadhesion to prolong ocular residence time and exploit paracellular pathways [56]. Furthermore, their highly tunable internal architecture readily accommodates complex biologics; specifically, engineered poly(lactic-co-glycolic acid) (PLGA) nanocarriers have been designed to achieve robust encapsulation and prolonged release of lactoferrin (LF), a strategy investigated for managing keratoconus-associated inflammation [57,58]. This polymeric efficiency extends directly to standard cross-linking agents. For instance, NPs synthesized from 2-hydroxypropyl-β-cyclodextrin (HP-β-CD) feature a unique hydrophobic-core and hydrophilic-shell architecture that effectively complexes with riboflavin. When co-formulated with chemical enhancers such as EDTA and trometamol to relax epithelial tight junctions, these NPs function as highly efficient riboflavin shuttles. Evaluated in ex vivo human corneas against the standard epi-off Dresden protocol, this formulation achieved a 46% overall increase in Young’s modulus but delivered nearly 50% less riboflavin to the mid-to-posterior stroma, highlighting a persistent challenge in deep tissue penetration [59].

Emerging as versatile in vivo preclinical candidates, nanocomposite and nanostructured hydrogels shift the ocular delivery paradigm from discrete nanoparticles to bulk biomimetic networks, providing a macroscopic environment that effectively recapitulates the corneal extracellular matrix (ECM) [60]. Capitalizing on the structural tunability of these platforms, researchers have engineered sutureless bioadhesive patches by co-assembling gelatin methacryloyl (GelMA), type I collagen, and bi-functional nanomicelles composed of Pluronic F127 diacrylate and aldehyde-modified Pluronic F127. By integrating these nanoscale building blocks into a macroscopic gel, this nanocomposite rapidly seals deep stromal defects with exceptional mechanical toughness upon UVA-mediated cross-linking. Evaluated in in vivo rabbit defect models, this nanocomposite successfully sealed deep stromal wounds with a burst pressure exceeding 400 mmHg and supported complete re-epithelialization within 14 days [61]. Rather than functioning as pre-formed surgical implants, alternative nanostructured hydrogel systems exploit physiological triggers to maximize non-invasive drug retention. Thermosensitive formulations utilizing Transcutol P and Pluronic F-127, which inherently self-assemble into micellar nanostructures, undergo rapid in situ gelation upon contact with the warm ocular surface near 31 °C, while the chemical enhancer simultaneously relaxes the epithelial tight junction. When evaluated utilizing an in vivo rabbit model comprising three animals per group, this phase transition prolonged residence time and generated a corneal stress of approximately 0.06 MPa at 10 percent strain. This mechanical reinforcement successfully outperformed established commercial benchmarks, demonstrating superior stiffening efficacy relative to both the transepithelial MedioCROSS TE and de-epithelialized MedioCROSS D reference formulations. Nevertheless, the cytotoxicity associated with higher Transcutol P concentrations highlights the critical need to balance biomechanical gains with cellular safety prior to clinical translation [62].

#### 3.1.3. Highly Ordered and Structural Penetration Platforms

Functioning as early-stage in vitro formulation candidates, highly ordered nanostructures dictate photosensitizer flux through their precisely defined internal geometries and spatial architectures. Within this structural category, liquid crystalline nanoparticles (LCNPs) exploit specific lyotropic structural transitions. Mechanistically, their bicontinuous lipid-water networks promote fusogenic membrane interactions, theoretically extending ocular residence and sustaining drug release [63]. Through spontaneous self-assembly, early physicochemical evaluations of sponge-phase LCNPs consistently achieve an average particle size of approximately 145 nm and a mean encapsulation efficiency of 46% [64]. While optimizing monoolein purities into distinct cubosome and hexosome phases elevates encapsulation to 65%, water-soluble payload capacity remains constrained by excess free aqueous phases. Furthermore, lacking ex vivo permeation data and direct biomechanical benchmarking, their therapeutic readiness remains highly speculative [65].

Engineered as highly tunable preclinical platforms, nano metal–organic frameworks (Nano-MOFs) exploit immense surface areas and tunable porosity for stimulus-responsive drug delivery [66,67]. Exemplifying this structural precision, hibiscus-like zeolitic imidazolate framework-8 (ZIF-8) microspheres (6RF@ZIF-8 NF) assembled from distinct nanoflakes utilize a hydrophobic yet negatively charged surface to facilitate transepithelial permeation. Structurally, this unique flake-assembled geometry maximizes tissue contact and shortens riboflavin release pathways. When evaluated in vivo in rabbits, this formulation achieved full-thickness stromal penetration, yielding higher Young’s modulus under identical strain conditions and matching the 40 h collagenase resistance of the standard epi-off Dresden protocol [13]. Nonetheless, while demonstrating favorable acute biocompatibility without endothelial apoptosis, the long-term safety of this MOF, particularly regarding the degradation and tissue accumulation of Zn^2+^, warrants further investigation.

Emerging as highly tailorable zero-dimensional platforms, carbon quantum dots (CQDs) offer ultra-small dimensions and versatile surface chemistry for ocular delivery [68]. Specifically, a covalently linked N,S-CQD–riboflavin conjugate (−20.1 mV) accelerates iontophoresis-assisted permeation and amplifies reactive oxygen species (ROS) generation by narrowing the singlet-triplet energy gap. In vivo, this synergy allowed a brief 10 min instillation and a halved UVA exposure (15 min), yielding biomechanical stiffening and enzymatic resistance that surpasses the standard epi-off Dresden protocol. While acute safety is favorable, clinical translation necessitates ruling out long-term stromal accumulation [69].

Functioning as highly localized macroscopic delivery platforms, microneedle (MN) arrays bypass chemical permeation barriers, utilizing a physical micro-puncture mechanism to deliver photosensitizers with controlled spatial precision [70]. Advancing beyond passive diffusion, contemporary MN designs incorporate stimulus-responsive dispensing mechanics. For instance, photothermal-responsive arrays equipped with a poly(N-isopropylacrylamide)/graphene oxide (PNIPAM/GO) hydrogel core physically contract under near-infrared irradiation, actively pumping riboflavin deep into the stroma through porous tips. Evaluations in vivo confirmed that this active extrusion matched the biomechanical efficacy of an accelerated epi-off baseline (4.32 J/cm^2^) while enabling complete epithelial recovery within 24 h [71]. However, the long-term ocular biocompatibility of GO components necessitates rigorous validation prior to human application. Furthermore, this solid-state architecture inherently supports topographical customization, providing a structural basis to match the array curvature to patient-specific corneal profiles for targeted biomechanical intervention [72]. While this innovative approach demonstrates excellent clinical potential, the current payload capacity necessitates supplementary topical instillation, a step that introduces a potential risk of lateral photosensitizer diffusion. Addressing the fundamental requirement of ocular safety, dissolvable MNs formulated from silk fibroin methacrylate (SFMA) demonstrate promising preclinical utility. When specifically engineered with a 550 µm tip length and a 1% riboflavin payload, these systems achieve complete stromal saturation and leverage the polymer’s intrinsic reactivity to drive multiple cross-linking cascades with corneal collagen. This structural synergy yields in vivo biomechanical stiffness significantly superior to the standard accelerated epi-off protocol while maintaining local biocompatibility in preliminary evaluations [73]. Beyond serving merely as photosensitizer shuttles, advanced MNs platforms are currently being engineered to actively co-deliver oxygen, a synergistic strategy detailed in Section 3.2.

The advantages and limitations of the compounds described above are summarized in Table 1.

#### 3.1.4. Comparative Analysis of Delivery Routes: Balancing Efficacy, Safety, and Feasibility

Translating nano-enabled cross-linking from the laboratory to clinical practice requires a comprehensive evaluation of ocular drug delivery routes. Selecting the optimal modality means weighing therapeutic efficacy against clinical feasibility and safety. Historically, the standard epi-off Dresden protocol ensures high spatial precision of API delivery and bulk stromal saturation. However, this aggressive approach causes extensive epithelial trauma, elevating the risk of severe stromal injury, endothelial toxicity, and postoperative discomfort, which significantly reduces patient compliance [74].

To overcome the severe trauma associated with bulk debridement, emerging minimally invasive methods such as MN offer a refined alternative to achieve high therapeutic efficacy [70]. By creating microscopic conduits through the anatomical barrier, these platforms maintain the enhanced bioavailability and localized biomechanical reinforcement necessary for advanced, rapidly progressing ectatic diseases [71,72,73]. Importantly, MNs significantly mitigate the adverse effects of traditional surgery; preclinical evidence demonstrates that these micro-punctures undergo rapid epithelial restitution, preserving endothelial integrity and avoiding widespread stromal damage [71]. Nevertheless, because these platforms still involve physical tissue penetration, they inherently carry a slightly higher risk of localized micro-trauma compared to completely non-invasive topical drops.

To mitigate these physical risks, non-invasive eye drops, specifically the topical instillation of stimulus-responsive nanogels, prioritize clinical safety and patient acceptability [62]. Theoretically, by completely preserving the epithelial barrier, these routes minimize the risk of stromal injury and are anticipated to improve patient compliance, making them conceptually well-suited for early-stage ectasia or long-term disease management. Yet, this safety involves a clinical trade-off: their reliance on passive diffusion or mild electrical driving forces inherently limits the spatial precision of API delivery, often confining optimal drug concentrations to the anterior stroma [59]. To deepen penetration, recent strategies combine specific nanocarrier surface charges with electrical driving forces. For example, the negative charge (−20.1 mV) of N,S-CQD–riboflavin conjugate facilitates iontophoresis-assisted transport, achieving efficient transepithelial stromal drug delivery without epithelial damage [69].

Beyond clinical outcomes, the adoption of these routes depends heavily on specific translational regulatory hurdles. Generally, as technological complexity increases to overcome physiological barriers, translational difficulty also rises. While simple topical nanocarriers face standard Chemistry, Manufacturing, and Controls (CMC) evaluations, sophisticated platforms are classified differently. Specifically, drug-device combination products face additional regulatory scrutiny, requiring rigorous dual-approval validation for both the engineering mechanics (e.g., iontophoresis devices or MN arrays) and formulation compatibility. Ultimately, rather than representing competing technologies, these delivery routes should be viewed as complementary strategies occupying distinct positions along the continuum between patient convenience and therapeutic precision.

#### 3.1.5. Payload Pairing: Structural and Physicochemical Alignment with Diverse Biomolecules

While riboflavin remains the standard photosensitizer for therapeutic cross-linking, comprehensively managing corneal ectatic diseases increasingly requires multi-modal pharmacological interventions. Transitioning from simple hydrophilic small molecules to complex biologics necessitates explicitly matching the payload’s physical dimensions and stability with the nanocarrier’s inherent physicochemical attributes. For hydrophilic small molecules such as riboflavin or targeted LOX activators, achieving high encapsulation efficiency within lipid matrices represents a primary hurdle. Conventional liposomes face cargo leakage and restricted loading capacity due to limited aqueous core volumes [53]. Similarly, while NLCs exhibit favorable structural stability, achieving high encapsulation efficiency for hydrophilic payloads typically requires complex formulation designs such as ion-pairing strategies [75]. To bypass these lipid partitioning barriers, highly ordered platforms like liquid crystalline nanoparticles (LCNPs) or zeolitic imidazolate frameworks (ZIF-8) leverage precise internal geometries and porous networks to improve loading and retention [13,64]. Alternatively, zero-dimensional carbon quantum dots (CQDs) utilize direct covalent esterification to bypass physical entrapment limits entirely, significantly enhancing stromal payload delivery while preventing cargo leakage [69].

Expanding beyond photochemistry, while specific clinical treatments for keratoconus utilizing siRNA or miRNA remain unestablished, these gene-modulating payloads demonstrate significant therapeutic potential across broader corneal applications [76,77]. However, these polyanionic macromolecules exhibit poor spontaneous membrane permeability and high susceptibility to enzymatic cleavage, strictly requiring nanocarriers that balance structural protection with charge matching. Cationic NLCs address this by utilizing positive surface charges to facilitate electrostatic condensation and enhance intracellular delivery, with endosomal escape driven by carrier-induced membrane destabilization [78]. Nevertheless, this positive surface charge represents a severe clinical constraint; excessive cationic density induces acute corneal epithelial apoptosis and endothelial toxicity, requiring rigorous zeta-potential titration during formulation. Alternatively, functionalized Nano-MOFs offer a distinct topological advantage by encapsulating fragile nucleic acids within their crystalline frameworks, thereby providing a robust steric shield against premature enzymatic degradation [79]. Similarly, peptide drugs (e.g., P2 or RGD sequences) and therapeutic proteins (e.g., LF or MMP inhibitors) necessitate carrier matrices that preserve fragile tertiary conformations against proteolytic degradation. Polymeric architectures, specifically PLGA nanoparticles and nanostructured/nanocomposite hydrogels, represent well-established platforms for protein encapsulation and sustained release [80,81]. These biomimetic networks provide highly tunable internal microenvironments that physically shield complex biopolymers from premature cleavage while mimicking the native corneal extracellular matrix to achieve prolonged therapeutic efficacy.

At the extreme spectrum of molecular weight, intact therapeutic antibodies present formidable translational hurdles. Due to their massive physical dimensions, standard diffusion-based nanocarriers generally exhibit limited stromal penetration across intact anatomical barriers. Consequently, antibody delivery into the stroma traditionally relies on direct barrier-disruptive interventions, such as conventional intrastromal microinjections. While advanced macroscopic delivery platforms, including stimulus-responsive or dissolvable MNs [82], are emerging as experimental alternatives to physically depositing antibodies directly into target tissues, standardized non-invasive protocols for antibody transport into the central optical zone remain unestablished. Ultimately, the successful translation of nano-enabled corneal therapies dictates that a carrier’s internal morphology, loading mechanism, and surface electrostatics must be explicitly governed by the specific physical and biological traits of its intended payload.

### 3.2. Overcoming Stromal Hypoxia: In Situ Oxygen-Generating Nanomaterials and Oxygen-Carrying Microneedles

The transition from standard to A-CXL is fundamentally bottlenecked by rapid stromal oxygen depletion, which prematurely quenches the highly efficient Type II photochemical cascade. Conventional physical strategies to mitigate this hypoxic environment, such as pulsed UVA irradiation or external oxygen-enriched chambers, depend on slow passive diffusion and have consequently failed to yield significant biomechanical improvements [33,38]. To actively reverse this metabolic limitation, promising advanced preclinical oxygen-self-supplying nanoplatforms shift the intervention from external delivery to in situ generation. A premier example is the use of graphitic carbon nitride quantum dots (g-C3N4 QDs). These nanomaterials leverage their intrinsic photocatalytic properties to continuously split stromal water and produce oxygen directly within the stroma upon 365 nm UVA irradiation. This internal replenishment sustains a robust yield of ROS even under high-fluence A-CXL conditions, effectively circumventing the anatomical diffusion barrier. Specifically, in vivo assessments in rabbits demonstrated that this in situ oxygenation under a 9 mW/cm^2^ accelerated protocol yielded corneal biomechanical stiffness and enzymatic resistance comparable to the standard epi-off Dresden protocol, significantly outperforming conventional accelerated treatments [14]. Furthermore, while short-term evaluations indicated remarkable preservation of endothelial cell density and favorable systemic biocompatibility, this intervention still relies on invasive epithelial debridement, and the long-term local clearance of these 5–8 nm QDs from the avascular stroma warrants further investigation.

Alongside in situ photocatalytic generation, physical oxygen-carrying systems present a viable alternative for mitigating stromal hypoxia. Recently, oxygen-releasing riboflavin MNs (O_2_RF@MNs) were engineered with a rapidly dissolving hyaluronic acid and polylactic acid matrix to simultaneously bypass the epithelial barrier and deliver oxygen directly to the deep stroma. By embedding perfluorodecalin nanoemulsions within this array, these MNs release pre-loaded oxygen via rapid physical dissolution within a narrow thirty-second window. When evaluated in vivo in rabbits, this dual-delivery approach achieved a corneal Young’s modulus 207% greater than the standard accelerated epi-off protocol, while accelerating epithelial healing and reducing the expression of pain-related cytokines [83]. Despite these excellent preclinical outcomes, the translation of this platform remains constrained by its extremely brief release window and strict requirement for oxygen-rich, light-shielded storage, presenting challenges for scalable manufacturing and extended shelf stability.

### 3.3. Bypassing Corneal Thickness Constraints: Visible-Light Paradigms and High-Precision Photonic Strategies

#### 3.3.1. Exploratory Paradigms: Visible-Light Photosensitizers, Hyperoxic Optimization, and Theoretical Nano-Amplifiers

To circumvent the inherent phototoxicity of conventional UVA irradiation, a highly speculative yet conceptually promising paradigm involves shifting activation thresholds into the safer visible-light spectrum. At the proof-of-concept in vitro level, conjugating visible-light-responsive Ruthenium(II) (Ru(II)) complexes with gold NPs creates a synergistic photocatalyst that drives type I collagen cross-linking via an oxygen-independent electron transfer mechanism [84]. Translating this photochemical mechanism to ocular models, ex vivo bovine cornea studies evaluating the free, non-nano Ru(II) complex under 430 nm blue light demonstrated a storage modulus reaching 190.48 kPa within 10 min, significantly outperforming the equivalent temporal benchmark of a customized riboflavin/UVA baseline [85]. Subsequent in vivo assessments in rat models confirmed that this non-nano complex mitigated cellular apoptosis and enabled complete epithelial regeneration by day 6, outperforming a low-dose UVA baseline (0.9 J/cm^2^) [86].

Alongside these blue-light-responsive complexes, Rose Bengal and green light cross-linking (RGX), which utilizes Rose Bengal activated by 532 nm irradiation, represents another prominent visible light alternative. Initial ex vivo benchmarking demonstrated that a 12 min RGX protocol (150 J/cm^2^) increased average stromal stiffness 3.8-fold and Young’s modulus 4.4-fold relative to untreated controls. While these biomechanical gains were lower than the 5.5-fold stiffness and 8.5-fold Young’s modulus increases elicited by the standard riboflavin/UVA Dresden protocol, the mechanical strengthening was strictly confined to the anterior 100–120 μm, thereby presenting a safer option for thin corneas [87]. Mechanistic evaluations confirmed that while this process is predominantly oxygen-dependent via singlet oxygen generation, it co-existed with an oxygen-independent electron-transfer pathway. Crucially, supplementing the formulation with electron donors like arginine can bypass the depth-dependent oxygen diffusion limit, enabling effective stromal stiffening even under anoxic conditions and lowering the energy thresholds required for deep tissue photobleaching [88]. Building on this metabolic framework, a recent in vivo rabbit study investigated whether actively supplementing the ocular surface with a hyperoxic environment (95% O_2_) could further elevate the therapeutic yield. This hyperoxic intervention achieved greater improvements in Young’s modulus and a more prolonged collagenase digestion time compared to the normoxic control group. Trypan blue/alizarin red and TUNEL staining verified endothelial safety despite transient airflow-induced thinning below the level of the normoxic control group. This progression from ex vivo mechanistic dissection to in vivo hyperoxic optimization supports visible-light cross-linking for thin corneas, though standardized delivery interfaces remain a prerequisite for clinical translation [89].

For therapeutic paradigms that retain UVA activation, minimizing irradiation exposure without sacrificing the biomechanical yield remains a critical priority. In this context, titanium dioxide (TiO_2_) NPs could be envisioned as a highly speculative class of UVA amplifiers. While their robust chemical stability and well-established photoresponsive properties have been well-documented in other non-ophthalmic fields [90,91], their application in corneal cross-linking remains an entirely theoretical extrapolation. Under UVA illumination, TiO_2_ NPs efficiently absorb and convert photonic energy, functioning simultaneously as a physical UVA shield and a photocatalytic generator of the ROS [91,92]. However, as explicitly demonstrated by their ROS-mediated cytotoxicity in human skin cells [92], extrapolating this mechanism to the cornea carries significant safety risks. Consequently, while incorporating such amplifiers could theoretically maximize the ROS yield during ultra-short UVA exposures, this concept remains purely conjectural and is currently far from clinical readiness.

#### 3.3.2. High-Precision Photonic Strategies

Although functioning as a purely physical photonic modality rather than a nanomaterial, two-photon corneal cross-linking (2P-CXL) conceptually mirrors the underlying logic of targeted nanocarriers: achieving strictly confined and depth-controllable interventions, a feature crucial for the safe cross-linking of thin corneas. To elevate the spatial resolution of conventional cross-linking, this paradigm fundamentally redefines photochemical control by confining the reaction exclusively to the microscopic focal volume of a femtosecond laser. As a highly speculative candidate for human applications, 2P-CXL harnesses near-infrared wavelengths (760–800 nm) for nonlinear optical activation. Within compressed type I collagen hydrogels, this highly localized three-dimensional (3D) modulation achieved biomechanical stiffening equivalent to the standard Dresden protocol [93]. Confocal Brillouin microscopy mapping in ex vivo bovine corneas further confirms this spatial precision, demonstrating a localized Brillouin frequency shift of 0.3–0.6 GHz that matches the efficacy of the standard Dresden protocol but remains restricted to the targeted focal volume [94]. When directly benchmarked against the standard Dresden protocol utilizing ex vivo human corneal stromal lenticules, micro-targeted 2P-CXL yielded superior elastic modulus improvements under physiological strains and comparable enzymatic resistance [95]. Independent optimization studies on similar human lenticular models corroborated this profound local effect, recording tissue modulus increases of up to 296% without inducing substantial optical transparency loss [96]. The primary hurdle for clinical translation lies in its slow scanning throughput. Because current systems rely on 1 kHz laser architectures, treating a minor 14 mm^2^ zone frequently requires over twenty minutes, keeping this technology far from near-term therapeutic readiness.

Beyond therapeutic activation, the two-photon excitation principle also drives high-resolution, 3D microscopy for noninvasive post-operative assessment, as demonstrated in in vivo rabbit CXL models utilizing tissue autofluorescence and second harmonic generation [97]. Conventional clinical evaluations of cross-linking efficacy typically necessitate over a month of passive observation. Two-photon microscopy (TPM) directly overcomes this temporal limitation; investigations utilizing ex vivo human corneas confirm that TPM can deliver structural and metabolic feedback via autofluorescence intensity and lifetime shifts as early as two hours post-procedure [98]. By seamlessly combining these structural and functional insights, two-photon imaging (TPI) emerges as a powerful investigational tool for real-time metabolic tracking and high-fidelity corneal evaluation [99]. However, its routine clinical translation remains impeded by high equipment costs, the necessity for contact interfaces, and a current lack of ophthalmic clinical certification.

**Figure 1:**
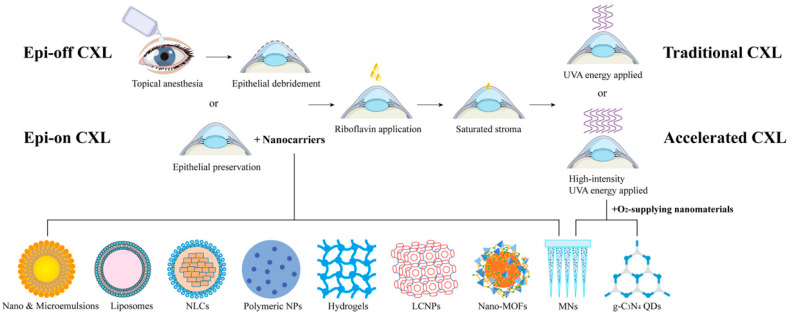
Nanotechnology-enabled modalities for corneal cross-linking (CXL). Traditional epi-off CXL requires epithelial debridement for riboflavin instillation. The lower sequence presents the nano-enhanced epi-on approach, utilizing diverse nanocarriers to drive transepithelial riboflavin permeation while leaving the epithelial barrier intact. On the right, the irradiation phase contrasts traditional CXL with accelerated CXL. Notably, the accelerated protocol integrates oxygen-supplying nanomaterials to mitigate stromal hypoxia induced by high-fluence irradiation. Abbreviations: CXL, corneal cross-linking; g-C_3_N_4_ QDs, graphitic carbon nitride quantum dots; LCNPs, liquid crystalline nanoparticles; MNs, microneedles; Nano-MOFs, nano metal–organic frameworks; NLCs, nanostructured lipid carriers; NPs, nanoparticles; UVA, ultraviolet A.

Table 2 correlates the clinical limitations of conventional CXL outlined in Section 2 with the nano-enabled solutions discussed in Section 3, detailing how specific nanomaterial mechanisms overcome targeted pathological barriers. Alongside this, Figure 1 illustrates the integration of these advanced platforms into the clinical CXL workflow.

### 3.4. The Pharmacological Landscape: Expanding APIs Beyond Riboflavin

Although riboflavin-mediated photochemistry mechanically reinforces the corneal stroma, keratoconus progression is increasingly recognized as a multifactorial process involving ECM degradation, impaired endogenous cross-linking capacity, oxidative stress, and localized inflammatory responses [100]. Transitioning toward comprehensive disease management requires expanding the pharmacological repertoire. However, the application of these alternative APIs in keratoconus remains investigational, and their clinical translation is frequently hindered by unfavorable physicochemical profiles. Consequently, specific nano-formulations represent a crucial prerequisite for achieving sufficient ocular bioavailability, thereby enabling rigorous evaluations of their independent pharmacological value, as well as their potential to function synergistically with standard cross-linking interventions.

Targeting the pathological imbalance between ECM synthesis and degradation represents a primary pharmacological avenue, particularly through the inhibition of upregulated proteolytic enzymes such as MMP-9 [101]. Pharmacological adjuncts like tetracycline derivatives (e.g., doxycycline) have demonstrated anti-collagenolytic effects in related ocular conditions, offering a theoretical rationale for repurposing them in ectasia management [102]. However, the topical ophthalmic delivery of APIs like doxycycline presents inherent formulation challenges. The extreme aqueous insolubility of its free base (molecular weight [MW] ≈ 444 Da) precludes direct incorporation into conventional aqueous drops, whereas its water-soluble salt derivatives (e.g., doxycycline hyclate) still suffer from rapid tear washout and limited stromal bioavailability. Encapsulation within lipid- or polymer-based nanocarriers can protect these labile molecules from premature degradation while prolonging precorneal residence and sustaining local drug release, thereby enhancing corneal tissue exposure and enabling more reliable evaluation of their anti-collagenolytic efficacy in vivo [103,104].

Parallel to inhibiting enzymatic degradation, pharmacologically upregulating endogenous cross-linking pathways offers a biomimetic alternative to UVA-mediated photochemistry. LOX, a copper-dependent enzyme, catalyzes the oxidative deamination of lysine residues to stabilize collagen fibrils [105]. Yet, the topical delivery of candidate LOX-modulating agents or their essential copper-based cofactors is challenged by unfavorable pharmacokinetic and safety profiles. In particular, freely soluble copper ions lack effective ocular retention and are readily eliminated by precorneal clearance mechanisms, whereas the narrow therapeutic window of transition metals raises concerns regarding dose-dependent ocular toxicity [106]. To address this, the encapsulation of these cofactors within sustained-release platforms, such as mesoporous silica nanoparticles or injectable hydrogels, has been proposed as a strategy to prolong local exposure [107,108]. This sustained delivery provides the foundation for exploring LOX modulation either as an independent biomechanical therapy or as a synergistic adjunct to traditional UVA cross-linking.

Finally, addressing the localized inflammatory and oxidative stress pathways that exacerbate ECM remodeling requires specialized microenvironmental modulation. This involves agents ranging from highly lipophilic immunomodulators (e.g., cyclosporine A, MW ≈ 1202 Da) to high-molecular-weight multifunctional biologics (e.g., LF, MW ≈ 80 kDa) [109,110]. Addressing their distinct delivery hurdles requires tailored nano-encapsulation strategies. For hydrophobic molecules such as cyclosporine A, partitioning them into nanocarrier hydrophobic domains can effectively overcome poor aqueous solubility and enhance ocular bioavailability [111]. Conversely, large biologics such as LF are constrained by limited tissue accessibility and susceptibility to proteolytic degradation; encapsulating these fragile macromolecules within PLGA nanoparticles or nanocomposite hydrogels can physically shield them from enzymatic cleavage while prolonging local retention and sustaining therapeutic exposure [58]. Ultimately, by overcoming the inherent physicochemical constraints of these diverse APIs, such targeted nanocarrier platforms facilitate the rigorous translation of these multi-targeted concepts into comprehensive ectasia management strategies.

Drawing together the diverse delivery strategies and structural payload alignments discussed above, Table 3 maps out the optimal pairings between specific nanocarrier platforms and emerging therapeutic agents, highlighting their delivery routes and mechanistic synergies.

## 4. Challenges and Future Directions

### 4.1. Challenges

#### 4.1.1. Biological and Toxicological Hurdles

As noted above, the application of nanomaterials to CXL for keratoconus is driving a paradigm shift. Intelligent design endows these platforms with multiple functions, opening new prospects in drug delivery and personalized therapy [112]. Despite these promising advancements, realizing the full clinical potential of these novel therapeutic modalities necessitates comprehensive preclinical evaluations of their intrinsic safety, efficacy, and long-term toxicity profiles. A critical pharmacological hurdle is the precise modulation of ROS. While robust ROS generation is essential for effective collagen cross-linking, an uncontrolled or excessive ROS burst can induce severe oxidative stress, triggering unintended apoptosis in healthy stromal keratocytes and the vulnerable corneal endothelium [113,114]. Moreover, the metabolic fate and clearance pathways of non-biodegradable nanomaterials within the avascular corneal stroma remain largely unelucidated, raising profound concerns regarding long-term stromal accumulation [115,116,117]. Furthermore, specific nanocarrier attributes such as cationic surface charges or the degradation byproducts of polymeric systems can present distinct cytotoxicity profiles. Beyond the materials themselves, formulating these nanocarriers for effective stromal penetration often involves conventional permeation enhancers like Transcutol P, whose toxicity is strictly dose-dependent, posing potential cytotoxic risks at high concentrations [55]. Conversely, while minimally invasive platforms like microneedles bypass these chemical formulation barriers, they introduce alternative safety liabilities, notably the inherent risk of physical micro-trauma and epithelial micro-injury. Consequently, robust longitudinal safety data remain largely absent in the current literature. To facilitate successful clinical translation, future research should ideally incorporate comprehensive long-term safety considerations, with a particular focus on elucidating ocular clearance mechanisms and precise ROS dosimetry. Table 4 provides a comprehensive risk-benefit assessment of the primary nanoplatforms, systematically weighing these inherent toxicological concerns against their therapeutic advantages.

#### 4.1.2. Translational and Regulatory Barriers

Most nanomaterials discussed in this review remain at the preclinical stage and require further bench-to-bedside translation. Currently, the bulk of efficacy and biocompatibility data is derived from in vitro cell cultures, ex vivo animal corneas (e.g., bovine or porcine models), and in vivo small animal studies. While frequently used in vivo rabbit models share a comparable central corneal thickness with humans, they do not fully replicate the dynamic tear turnover rates, unique immune microenvironments, and specific microstructures like Bowman’s layer found in the living human eye.

Beyond these physiological differences, a notable clinical evidence gap persists. While existing preclinical studies provide valuable preliminary data on epithelial healing, corneal haze, and endothelial safety, evaluating the true advantage of a nanoplatform requires functional clinical metrics rather than relying solely on laboratory measurements of stromal stiffening or riboflavin penetration. Essential outcomes, including visual acuity recovery, refractive stability, subjective pain scores, and long-term ectasia progression control, are largely absent in the current literature.

Additionally, the heterogeneity in experimental designs complicates robust cross-study comparisons. The existing literature contains widely disparate UVA irradiation parameters, unstandardized animal models, and varying dosing regimens. Ultimately, the absence of direct human data and the lack of standardized clinical outcome reporting represent major barriers to transitioning these nano-enabled therapies from bench to bedside.

To bridge this biological gap and initiate rigorous human clinical trials, securing a reliable supply of strictly standardized therapeutic agents is essential. Yet, transitioning from small laboratory batches to large-scale industrial production remains one of the most challenging steps in nanomedicine development. Complex synthesis protocols and physicochemical variability complicate manufacturing, leading to high costs and batch-to-batch inconsistency. Meeting standard ophthalmic requirements for pH, osmolarity, and pharmacopoeial sterility requirements adds further difficulty. Sterilization is particularly challenging: thermal autoclaving often degrades nanostructures or causes payload leakage, while standard sterile filtration is unsuitable for large or viscous formulations. Additionally, ensuring long-term shelf stability without particle aggregation, phase separation, or drug degradation remains difficult.

For clinical translation, platforms based on clinically established materials such as liposomes, nanoemulsions, and PLGA nanoparticles are the most viable candidates for Good Manufacturing Practice (GMP) scale-up. Conversely, complex designs like Nano-MOFs face major scaling limitations. To overcome these manufacturing hurdles, robust quality control frameworks are imperative to ensure product consistency and safety [118]. Advanced manufacturing techniques, such as microfluidic-chip synthesis, may enable more controllable and reproducible production of nanocarriers while reducing batch-to-batch variation [119].

Beyond manufacturing hurdles, the absence of clear and consistent regulatory guidance further stalls clinical translation. Although agencies like the FDA and EMA have established preliminary frameworks, significant hurdles persist. These include the lack of unified definitions, complex physicochemical characterization requirements, and unstandardized biosafety assessment protocols [120]. Moving forward, refining these regulatory landscapes with standardized, ophthalmic-specific guidelines will be essential to facilitate the safe and efficient clinical application of these nano-enabled therapies.

### 4.2. Future Directions: Towards a Theoretical “Sense–Decide–Act” Framework

Conventional “one-size-fits-all” CXL protocols often yield unpredictable clinical outcomes due to substantial inter-individual variability. Initial attempts at personalization largely focused on single-variable adjustments, such as modifying treatments based on keratometry thresholds [121] or adapting UVA doses for ultrathin corneas using the Sub400 protocol [122]. Concurrently, microfabrication advances now allow for spatially customized drug delivery; notably, MN arrays can be geometrically tailored to match a patient’s specific corneal topography [72].

Although such structural customizations are essential, realizing true personalization is bottlenecked by the scarcity of specialized expertise in corneal ectasia and the analytical burden of integrating multidimensional preoperative data. Bridging this gap necessitates an intelligent system that can replicate expert clinical judgment to generate spatially tailored therapeutic parameters. Despite the urgent need for such artificial intelligence (AI)-directed therapy, current AI applications in keratoconus remain largely confined to early diagnosis and classification, drawing on multimodal data such as tomography, clinical risk factors, and AI-derived biomechanical indices [123,124,125]. Although pioneering mechanistic models, such as those developed by Hafezi et al., to predict post-CXL stiffening are emerging [126], shifting from AI-assisted diagnosis to AI-directed therapy remains a major challenge.

Furthermore, even with highly customized pre-operative planning, the dynamic nature of CXL surgery necessitates intraoperative adjustment. Achieving truly dynamically tunable therapy requires real-time theranostic feedback. Recent ex vivo studies and randomized clinical trials have shown that real-time fluorescence can successfully monitor corneal riboflavin concentration and predict CXL efficacy [127,128], positioning such theranostic systems as promising early translational candidates. However, tracking the photosensitizer alone is insufficient, as the true therapeutic endpoint is stromal mechanical stiffening. Consequently, integrating real-time in vivo biomechanical mapping via Brillouin confocal microscopy or optical coherence elastography (OCE) represents a crucial theoretical next step. While OCE has demonstrated the ability to dynamically track corneal biomechanical changes during CXL in ex vivo porcine corneas [129], it has not yet been incorporated as an active feedback regulator within the treatment platform.

**Figure 2:**
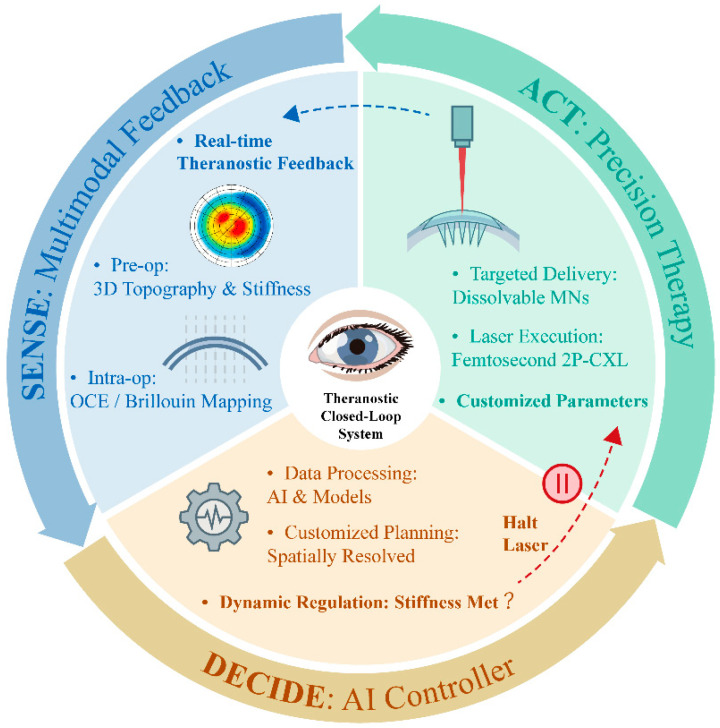
Concept of a personalized closed-loop theranostic system for keratoconus management. Multimodal inputs, including 3D topography and real-time OCE/Brillouin stiffness mapping (SENSE), are processed by an AI-driven controller (DECIDE) to generate customized therapeutic parameters. Treatment is executed via targeted riboflavin delivery using dissolvable MNs and spatially resolved femtosecond 2P-CXL (ACT). This dynamic feedback mechanism ensures automatic cessation of laser irradiation strictly upon reaching the predefined target stiffness. **Note:** The dashed blue arrow indicates the real-time theranostic feedback pathway that transmits intraoperative stiffness data back to the SENCE; the red pause symbol (II) marks the trigger point for automatic laser cessation when the predefined target stiffness is reached.

Looking toward the distant future, CXL is conceptually envisioned to evolve into a fully personalized, “sense–decide–act” closed-loop system (Figure 2). In this theoretical setup, a patient’s 3D corneal topography and baseline stiffness would act as multimodal inputs. AI algorithms would then process these to generate highly customized parameters for targeted photosensitizer delivery and spatially resolved laser execution. Intraoperatively, advanced nonlinear optical techniques like femtosecond laser-induced 2P-CXL would perform precise, 3D cross-linking following the spatially tailored delivery of riboflavin via optically transparent or dissolvable nanocarriers/MNs. Concurrently, integrated OCE or Brillouin modules would map biomechanics in real time. Once the target stromal stiffness is reached, the AI controller would halt the laser. However, this AI-integrated closed-loop system and 2P-CXL remain highly speculative concepts. Although presenting a theoretical framework for managing corneal ectatic diseases, these approaches are far from clinical use and primarily guide long-term research efforts.

## 5. Conclusions

The integration of nanotechnology into CXL represents a highly promising turning point in the management of keratoconus. By rationally engineering nanocarriers and photocatalysts, researchers can now provide innovative strategies to overcome the long-standing clinical barriers of conventional CXL, including epithelial resistance, stromal hypoxia, and endothelial vulnerability in thin corneas. Beyond enhancing drug delivery and photochemical efficiency, these nanomaterials serve as foundational elements for a broader therapeutic evolution. As we look to the future, the true potential of CXL lies in breaking free from “one-size-fits-all” paradigms. The integration of AI-assisted decision-making and dynamic biomechanical monitoring with customized nanomedicine outlines a theoretical visionary framework for a fully personalized, closed-loop treatment system. Clinical translation, however, hinges on resolving manufacturing bottlenecks, standardizing safety metrics, and establishing clear regulatory pathways. By rigorously navigating these translational hurdles, such advanced interdisciplinary strategies ultimately hold the potential to redefine the standard of care for keratoconus and other progressive corneal ectasias.

## Figures and Tables

**Table 1:** Comparative performance of different nanocarrier platforms in CXL.

Advanced Delivery Platforms	Delivery Efficiency	Biocompatibility	Current Research Stage	Key Advantage	Major Limitations
Nanoemulsions	High	Moderate (Surfactant irritation risk)	Preclinical (In vitro/Ex vivo)	Overcomes lipophilic epithelial barrier	Poor stability
Microemulsions	Moderate	Good	Preclinical (In vivo)	Sequential interfacial controlled release	Suboptimal sustained biomechanical stiffening
Liposomes	Low	Excellent	Preclinical (In vitro/Ex vivo)	High ocular safety	Low encapsulation efficiency
NLCs	High	Good	Preclinical (In vivo)	High payload capacity via disordered matrix	Unverified long-term epithelial safety
Polymeric NPs	High	Good (Polymer-dependent)	Preclinical (In vitro/Ex vivo)	Mucoadhesion and high structural plasticity	Insufficient deep stromal penetration
Nanocomposite and Nanostructured Hydrogels	High	Excellent	Preclinical (In vivo defect repair)	Biomimetic nanocomposite network	Enhancer-associated cytotoxicity
LCNPs	Moderate	Good	Preclinical (In vitro)	Bicontinuous lipid-water fusogenic networks	Constrained water-soluble payload
Nano-MOFs	Extremely High	Good	Preclinical (In vivo)	High riboflavin loading; targeted release	Constituent metal ion toxicity
CQDs	High (via iontophoresis)	Good	Preclinical (In vivo)	ROS amplification; halves UVA exposure time	long-term stromal accumulation risks
MNs	High	Good	Preclinical (In vivo)	Topographically customizable	Unverified long-term biocompatibility of complex composites

Abbreviations: NLCs, nanostructured lipid carriers; NPs, nanoparticles; LCNPs, liquid crystalline nanoparticles; MOFs, metal–organic frameworks; CQDs, carbon quantum dots; ROS, reactive oxygen species; UVA, ultraviolet A; MNs, microneedles.

**Table 2:** Mapping conventional CXL limitations to emerging nano-enabled solutions.

Clinical Limitation (Section 2)	Conventional Workaround	Advanced Interventions (Section 3)	Representative Nanomaterials and Exploratory Paradigms	Mechanism of Action
Epithelial Barrier	Iontophoresis Chemical enhancers	Transepithelial penetration	NLCs LCNPs Nano-MOFs CQDs MNs	Lipid partitioning Dynamic phase adaptation Surface charge and specific topology Electrostatic repulsion and ROS amplification Physical micro-puncture
Stromal Hypoxia (in A-CXL)	Pulsed UVA Supplemental O_2_	Intrastromal oxygen supply	g-C_3_N_4_ QDs O_2_RF@MNs	Photocatalytic O_2_ generation Physical pre-loaded O_2_ release
Thin Corneas (<400 µm)	Hypo-osmolar swelling CACXL EI-CXL	High-precision spatial confinement	2P-CXL RGX	Strictly confined 3D modulation Shallow anterior stromal confinement
Endothelial Vulnerability to UVA	Reduced exposure time	Visible-light shifting UVA amplification	Ru(II) complexes RGX TiO_2_ NPs	Shifting activation threshold to 430 nm blue light Shifting activation threshold to 532 nm green light ROS yield during ultra-short UVA exposure

Abbreviations: NLCs, nanostructured lipid carriers; LCNPs, liquid crystalline nanoparticles; MOFs, metal–organic frameworks; CQDs, carbon quantum dots; MNs, microneedles; ROS, reactive oxygen species; A-CXL, accelerated corneal cross-linking; UVA, ultraviolet A; QDs, quantum dots; RF, riboflavin; CACXL, contact lens-assisted corneal cross-linking; EI-CXL, epithelium-intact corneal cross-linking; 2P-CXL, two-photon corneal cross-linking; RGX, Rose Bengal and green light cross-linking; TiO_2_, titanium dioxide; NPs, nanoparticles.

**Table 3:** Multidimensional evaluation of nanocarrier platforms, ideal payload pairings, and delivery routes for advanced remodeling of ectatic corneas.

Nanocarrier Type	Ideal Payload Type	Preferred Delivery Route	Cross-Linking Mechanistic Synergy
Lipid-Based Systems (Liposomes, NLCs, LCNPs)	Small Molecules: Cyclosporine A, Doxycycline salts. Nucleic Acids: siRNA/miRNA.	Non-invasive: Topical instillation.	Epithelial Partitioning: Permeates the hydrophobic epithelium, enhancing solubility and bioavailability of lipophilic agents.
Polymeric NPs and Hydrogels (PLGA, Nanocomposites)	Small Molecules: Doxycycline salts, Cu^2+^. Peptides/Proteins: LF.	Non-invasive: Topical instillation/In situ gelation.	Sustained Residence and Shielding: Mucoadhesion prolongs tear film retention; polymeric matrices physically shield fragile biologics from enzymatic cleavage.
Rigid Nanoplatforms (Nano-MOFs, MSNs, CQDs)	Small Molecules: Riboflavin, Cu^2+^. Nucleic Acids: siRNA.	Non-invasive: Topical instillation.	Geometric Confinement: Bypasses lipid barriers by trapping hydrophilic payloads in porous networks; facilitates electrically driven trans-epithelial transport.
MNs	Small Molecules: Riboflavin. Antibodies: Intact therapeutic antibodies.	Minimally invasive: Stromal micro-penetration.	Physical Barrier Disruption: Breaches the epithelium for precise stromal deposition of diverse payloads from small molecules to massive biologics bypassing molecular weight limits.

Abbreviations: NLCs, nanostructured lipid carriers; LCNPs, liquid crystalline nanoparticles; siRNA, small interfering RNA; miRNA, microRNA; NPs, nanoparticles; PLGA, poly(lactic-co-glycolic acid); MOFs, metal–organic frameworks; MSNs, mesoporous silica nanoparticles; CQDs, carbon quantum dots; MNs, microneedles.

**Table 4:** Comprehensive ranking of nano-enabled CXL platforms: Primary benefits, safety liabilities, and translational maturity.

Nanoplatform	Primary Biomechanical/Therapeutic Benefit	Safety Liabilities and Clinical Risks	Translational Maturity and Industrial Feasibility
Nanoemulsions	Breaks lipophilic epithelial barrier; drives rapid transepithelial flux.	Surfactant-induced epithelial irritation; unverified stiffening efficacy.	In vitro/Ex vivo: Poor thermodynamic stability; sterilization may lead to phase separation.
Microemulsions	Dilution-triggered drug release; thermodynamically stable.	Hardening effect remains suboptimal compared to epi-off standard.	In vivo: High reproducibility via self-assembly; low cost; requires GMP scale-up optimization.
Liposomes	High ocular safety and excellent biocompatibility.	Fails to secure sufficient payload for stromal saturation.	In vitro/Ex vivo: Classic liposomal manufacturing is mature, but inefficient for this specific hydrophilic payload.
NLCs	Disordered matrix enables high payload capacity.	Dose-dependent cytotoxicity from chemical enhancers.	In vivo: Scalable synthesis; requires precise batch-to-batch quality control.
Polymeric NPs	Enhanced bioadhesion; prolonged retention; accommodates biologics.	Formulation-dependent burst release risks; potential byproduct cytotoxicity.	In vitro/Ex vivo: High structural plasticity; polymer-dependent sterilization
Nanocomposite and Nanostructured Hydrogels	Recapitulates extracellular matrix; seals stromal defects.	Highly biocompatible, but lacks direct transepithelial flux without enhancers.	In vivo: Viable for GMP sutureless patch production; shelf-life and sterile packaging challenges.
LCNPs	Bicontinuous channels provide immense surface area and high payload capacity.	Long-term stromal retention and in vivo CXL safety remain unverified.	In vitro: Scalable synthesis; structural phase highly sensitive to temperature/pressure, complicating batch control.
Nano-MOFs	Immense surface area; shortened release pathways via specific topology.	Long-term stromal accumulation risks; potential surface-charge toxicity.	In vivo: Intricate synthesis; poor scalability; high cost and batch variation.
CQDs	Drives rapid iontophoresis-assisted flux; amplifies ROS to halve UVA exposure time.	Potential long-term stromal accumulation.	In vivo: Low-cost precursors and scalable synthesis; requires rigorous purification optimization.
MNs	Direct intra-stromal dual-delivery; topographically customizable.	Inherent risk of mechanical micro-trauma and epithelial micro-injury.	In vivo: Biocompatible polymers (HA/SFMA) match ophthalmic standards; high replication cost and scalability bottlenecks.
Oxygen-Supplying QDs	Continuous in situ photocatalytic oxygen generation; reverses hypoxia.	Unelucidated stromal clearance and metabolic fate of inorganic dots.	In vivo: Complex purification; low reproducibility; strict regulatory definition barriers.
Visible-Light Photosensitizers	Shifts activation threshold to 430 nm/532 nm visible light; eliminates UVA phototoxicity.	Heavy metal (Ruthenium) clearance pathways and systemic toxicity unverified.	In vivo: High chemical stability; high material cost; lacks standardized clinical dosimetry.
TiO_2_ Amplifiers	Dramatically intensifies ROS yield; functions as a physical UVA shield.	Excess ROS burst triggers unintended endothelial and keratocyte apoptosis.	Conceptual: Low-cost inorganic synthesis; unverified ophthalmic compatibility and shelf life.

Abbreviations: GMP, good manufacturing practice; NLCs, nanostructured lipid carriers; NPs, nanoparticles; LCNPs, liquid crystalline nanoparticles; MOFs, metal–organic frameworks; CQDs, carbon quantum dots; ROS, reactive oxygen species; UVA, ultraviolet A; MNs, microneedles; HA, hyaluronic acid; SFMA, silk fibroin methacrylate; QDs, quantum dots; TiO_2_, titanium dioxide.

## Data Availability

No new data were created or analyzed in this study. Data sharing is not applicable to this article.
